# Yield and water productivity of rice as influenced by crop establishment and irrigation methods under temperate environment

**DOI:** 10.1038/s41598-025-09584-w

**Published:** 2025-08-12

**Authors:** Tasneem Mubarak, Intikhab Aalum Jehangir, Ashaq Hussain, Eajaz Ahmad Dar, Zahoor Ahmad Shah, Aabid H. Lone, Mohd Salim Mir, Salah El-Hendawy, Mohamed A. Mattar, Ali Salem

**Affiliations:** 1 Mountain Research Centre for Field crops, Sher-e-Kashmir University of Agricultural Sciences and Technology of Kashmir, Khudwani, Kulgam, 192102 India; 2 Sher-e-Kashmir University of Agricultural Sciences and Technology of Kashmir, Krishi Vigyan Kendra-Ganderbal, India 190006; 3 University of Florida, West Florida REC, Jay, Florida USA 32565; 4 Division of Agri. Extension, Faculty of Agriculture, Sher-e-Kashmir University of Agricultural Sciences and Technology of Kashmir, Wadura, Sopore, 193201 India; 5 Division of Agronomy, Faculty of Agriculture, Sher-e-Kashmir University of Agricultural Sciences and Technology of Kashmir, Wadura, 193201 Sopore India; 6 Department of Plant Production, College of Food and Agricultural Sciences, King Saud University, P.O. Box 2460, Riyadh 11451, Saudi Arabia; 7 Department of Agricultural Engineering, College of Food and Agricultural Sciences, King Saud University, P.O. Box 2460, Riyadh 11451, Saudi Arabia; 8 Structural Diagnostics and Analysis Research Group, Faculty of Engineering and Information Technology, University of Pécs, Pécs, 7622 Hungary

**Keywords:** Sustainable rice management, Sustainable water management, Resource conservation, Profitability, Temperate environment, Agroecology, Agroecology

## Abstract

This study compares direct-seeded rice (DSR) with transplanted rice under three different irrigation methods: conventional flooding, saturation, and alternate wetting and drying. The findings indicate that DSR outperforms transplanted rice in several key aspects. Specifically, DSR exhibited a greater number of tillers (635–650) and panicles (510–529) m^− 2^, along with lower spikelet sterility (9.9–10.8%). Grain yield ranged from 6.69 to 7.8 t ha^−1^ for DSR, surpassing that of transplanted rice, which yielded between 5.59 and 6.18 t ha^−1^. Moreover, DSR demonstrated 11–25% higher water productivity, highlighting its greater efficiency in water usage. Economic analysis revealed that DSR also offered superior returns, with a profit per rupee invested of 2.2, compared to 1.48 for transplanted rice. While irrigation method did not significantly impact growth or yield, conventional flooding led to a 28% reduction in water productivity compared to the saturation method and a 25% reduction compared to the alternate wetting and drying method. In terms of benefit-cost ratio, while the differences were modest, the saturation method recorded slightly higher values. Overall, the study indicates that adopting DSR with either the saturation or alternate wetting and drying irrigation methods can lead to higher yields, improved economic returns, and increased water productivity in temperate environments, positioning it as a more sustainable and efficient alternative to transplanted rice cultivation.

## Introduction

Rice (*Oryza sativa* L.) is grown over an area of 165 million ha across the world and is staple food for more than half of the global population^1^. More than 90% of rice supply comes from Asia^2^which makes this continent a leading contributor towards the food security of millions of people. India and China together produce 54% of the global rice with a production of 282.5 million metric tonnes^3^. In view of changing climatic conditions and receding water resources, the world’s largest staple food is facing serious challenges for sustainability. Availability of water^4^ and workforce^5^ are considered two major factors which could substantially impact rice production in future. Rice consumes a huge quantity of water and therefore is often referred as water guzzler. It is estimated that for the production of 1 kg of rice, 3000 to 5000 L of water are consumed^6^. Demand for rice is increasing due to increase in population and this is expected to increase the water requirement by 55% by 2050 globally^7^. Labour availability on the other hand is decreasing by 0.2% per annum in Asia^8^making it expensive day by day. Additionally, rice farming with conventional flood irrigation produces methane which is considered a potential greenhouse gas contributing to climate change^9^. These problems collectively pose a significant threat to rice production and may, therefore, impact the food security. In Kashmir valley situated in the northwest Himalayan region of India, rice is the staple food^10^. Traditionally rice is cultivated through flood irrigation method and canal irrigation is the primary source of irrigation. Studies indicate that water resources are shrinking in the valley^11^ and this may trigger shift from traditional rice farming to other crops and technologies which need less water. This will increase more dependency on outsourcing and imports of staple food, which also seems challenging in view of changing climatic and geo-political situations across the word. Sustaining rice farming therefore needs modifications in approach to cultivate it. Direct seeded rice (DSR) is emerging as a potential technology to address various issues related to rice farming. It not only saves water and labour but also reduces the risk of climate change. It also eliminates the chances of soil structure destruction occurring due to puddling under traditional rice farming. So far as the research on comparative performance of DSR and transplanted rice are concerned, some studies indicate that DSR yield may either match or exceed transplanted rice^12,13^. However, some other studies revealed contrary results^14^.

Along with efficient resource management, it is also important to sustain higher yields to meet the growing demand of food. Different water management practices are reported to impact the rice yield. Some studies show that alternate wetting and drying (AWD) is best practice in terms of yield and water productivity^15^while others reported that saturation method gave superior yield^16^. Previously some work on DSR has been conducted under Kashmir valley conditions but the comparative studies on transplanted and direct seeded rice under different irrigation methods are missing. With an objective to compare the performance of two establishment methods such as transplanting and direct seedling and three irrigation methods such as, conventional flooding, saturation and alternate wetting and drying, in terms of crop yield, water productivity and economics, the current study was undertaken.

## Materials and methods

### Site description

**Fig. 1:**
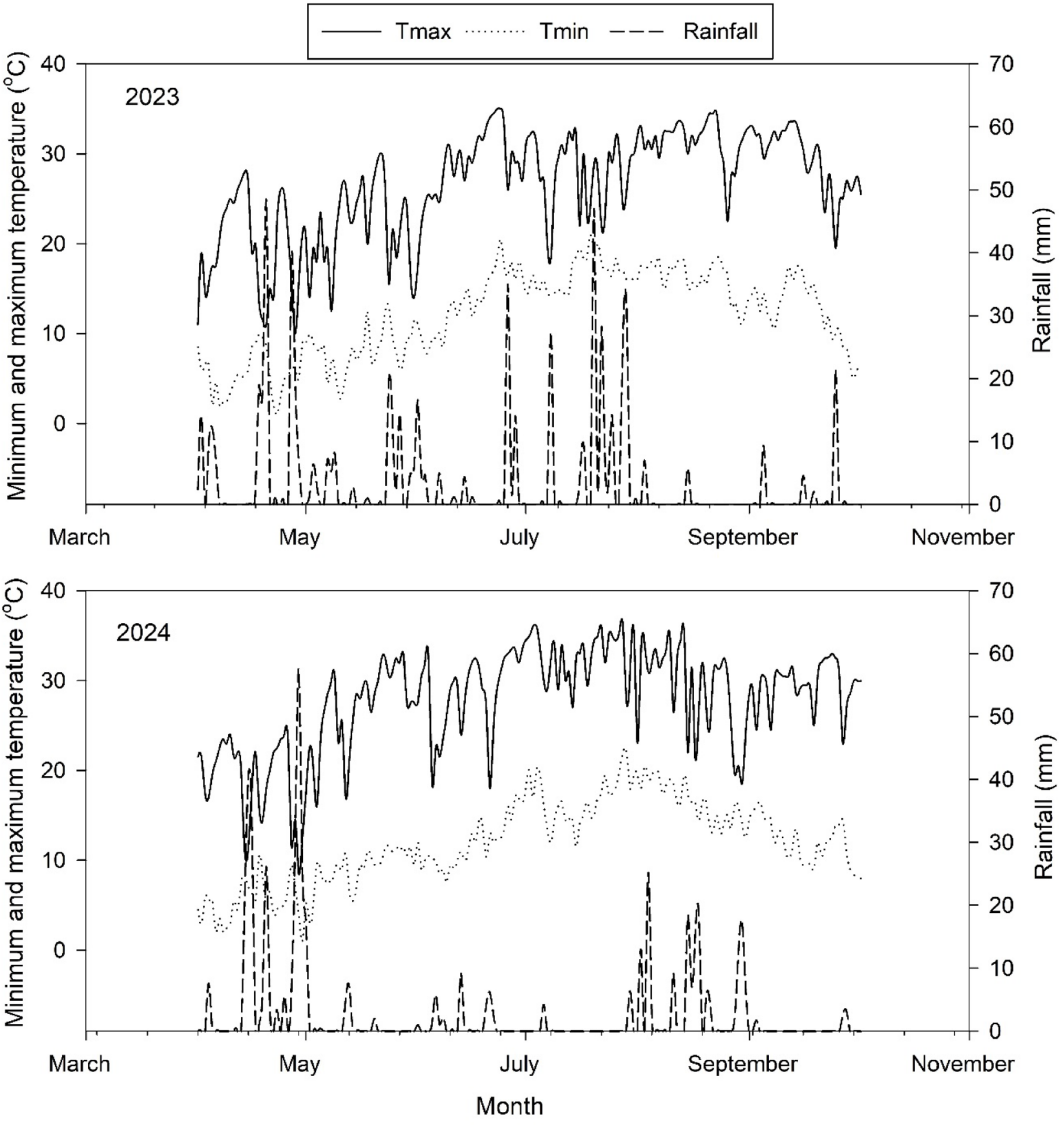
Weather data for the growing season of 2023 and 2024, including maximum (Tmax) and minimum (Tmin, °C) temperature and rainfall (mm).

Experiments were conducted in 2023 and 2024 at Mountain Research Centre for Field Crops (MRCFC), Sher-e- Kashmir University of Agricultural Sciences and Technology of Kashmir (33.723°N and 75.092 °E). Elevation of experimental site is 1590 m above mean sea level, and the location is characterized by temperate climatic conditions with mild summers and harsh winters. Minimum and maximum temperatures during crop seasons were 12.12 °C and 25.9 °C in 2023. In 2024, minimum and maximum temperatures were 13.4 °C and 29.3 °C (Fig. 1). Rainfall received in the two growing seasons was 647 mm and 244 mm, respectively.

Soil samples were collected from upper 20 cm depth before the start of experiment for physico-chemical analysis. The texture of the soil was determined by international pipette method^17^ and soil reaction by 1:2 soil-water suspension with Beckman’s glass electrode pH meter^18^. Estimations of available N, P and K in kg ha^− 1^ were done by applying alkaline potassium permanganate method^19, ^0.1 N sodium bicarbonate method^20^ and ammonium acetate method^18, ^respectively.

### Experimental design, treatment details and crop husbandry

Experiment was laid out in a split plot with establishment methods such as E_1_: transplanted rice and E_2_: DSR in main plots and irrigation methods such as I_1_: conventional flooding; I_2_: saturation and I_3_: alternate wetting and drying (AWD) in subplots. A detailed account of crop husbandry operations is given in Table 1. Soil of the experimental plot was brought to good tilth by one deep ploughing using a disc plough followed by two light harrowing and one planking. To check lateral movement of water from one plot to the other, a buffer of 0.5 m with compact earthen bunds of 30 cm height were made between the subplots receiving irrigation treatments. Similar buffers were also made between replications. Shalimar Rice-4, a locally bred high yielding variety, resistant to paddy blast was sown on 22 May in the nursery for transplanted rice and in main field for DSR during 2023. In 2024, sowing was done on 15 May for both transplanted rice (in nursery) and DSR (main field). Seed rate for transplanted rice was 60 kg ha^−1^ and for DSR it was 23 kg ha^−1^. In DSR seed was dibbled (3 seeds hill^−1^) at a spacing of 20 cm x 20 cm and in transplanted rice, one month old seedlings were transplanted in puddled plots at a geometry of 15 cm x 15 cm during both the years. A light irrigation was given after sowing of seed in DSR plots to induce germination.

A dose of 120:60:30 kg of N:P_2_O_5_:K_2_O was applied to transplanted as well as DSR during both the years. Fertilizers were used in the form of Urea, diammonium phosphate and muriate of potash, respectively. Full doses of P and K were applied as basal. Nitrogen was applied in three equal splits (basal at the time of sowing/transplanting, 1st top dose at maximum tillering stage and second top dose at panicle initiation stage). Weeds were managed by tillage and application of herbicides. Herbicides were applied with the objective of minimizing weed populations, following the standardized recommendations for each establishment method as outlined by MRCFC-SKUAST(K). In transplanted rice, Pretilachlor (6%) + Pyrazosulfuron ethyl (0.15%) (Eros) was applied 2 and 3 days after transplanting during 2023 and 2024, respectively. For DSR, Triafamon (20%) + Ethoxysulfuran (10%) (Council) was applied as early pre-emergence herbicide, 20 days after sowing^2^.

Although both canal irrigation, which is commonly adopted in the region, and tube-well irrigation were available, irrigation requirements as per treatments were met through tube-well with predetermined discharge rate. Flux variability was checked, and flow was found consistent during each season. In AWD, field water tubes were inserted for measuring water level. Tubes were made locally and used for observation and irrigation management as per the guidelines available at Rice Knowledge Bank of International Rice Research Institute, Philippines^21^. Since no significant disease and pest incidence was observed during both the years of study, no fungicide or insecticide was applied.

### Data recording

Plant height and tiller count were recorded at active tillering, panicle initiation (PI), heading and physiological maturity growth stages during both the years. Plant height and tiller count were recorded from 10 randomly selected and tagged hills from each plot and then converted into mean plant height and tiller number m^−2^. Ten Tagged plants were used to note 50% heading stage applying the method given by^22^. Yield attributing traits including ears m^−2^, grains panicle^−1^ and 1000 grain weight (g) were also recorded from 10 tagged hill from each plot. Grain and straw yields were recorded after harvesting and drying the crop. Crop was harvested when 95% of spikelets turned yellow. It was then tied in bundles and kept for drying until the threshing was done when moisture in grain was around 14%. Spikelet sterility was calculated from 10 randomly selected panicles using the formula given below^23^.

$$\:Spikelet\:sterility\:\left(\%\right)=\frac{Number\:of\:sterile\:spikelet}{Number\:of\:spikelets\:per\:panicle}\times\:100$$

Total water input including the water applied through irrigation and water received through rainfall (Table 2). Water applied through irrigation was measured based on predetermined discharge rate (943 cm3 sec^−1^) of tube-well near the experimental site. Rainfall data was obtained from the automatic weather station of MRCFC. Water productivity (WP) was calculated by using following formulae^24^.

1 $$\:WP\:\left(kg\:{m}^{-3}\right)=\frac{Grain\:yield\:\left(kg\right)}{Water\:input\:\left({m}^{3}\right)}$$

Experimental plot was frequently observed at 2 days interval to record attainment of different phenological stages, including maximum tillering, PI, 50% flowering and physiological maturity^25^.

Different meteorological indices were determined based on the data collected from the automatic weather station of MRCFC. Cumulative GDDs were calculated by summing the daily mean temperature above a base temperature (10 °C for rice[2])) and expressed in degree day. GDD was determined by using formula given below^26^

2 $$\:GDD=\frac{Maximum\:temperature+Minimum\:temperature}{2}-Base\:temperature$$

Multiplication of GDD and hours of bright sunshine (BSH) represents the Heliothermal units (HTU) for that day. Total HTU for the phenophases were computed according to the equation used by^26^

3 $$\:HTU=\sum\:\left({GDD}\times\:BSH\right)$$

Amount of dry matter produced per unit of GDD indicates the heat use efficiency. In our study HUE was calculated by using the formula given below^26^

4 $$\:HUE=\frac{Grain\:yield}{GDD}$$

Benefit cost ratio (B: C), which determines relationship between net returns and cost of cultivation, was calculated using formula given below:5

where, ₹= Indian rupee.

Carbohydrate equivalent (t ha^− 1^) was determined by multiplying yield with carbohydrate content in rice (78.05) as suggested by^27^.

### Data analysis

Each year data recorded on various growth, yield attributing traits and crop yield were subjected to analysis of variance using OPSTAT^®^ statistical software^28^. Means were separated based on the critical difference (CD) test at 5% level of probability (α = 0.05). Graphs were designed using SigmaPlot 15.0 (Grafiti LLC, CA; https://grafiti.com/sigmaplot-detail/). Curve fitting was done through dynamic curve fit using SigmaPlot 15.0.

**Table 1:** Details of crop husbandry during the rice growing seasons of 2023 and 2024 at mountain research center for field crops-SKUAST-Kashmir.

Crop husbandry operation	2023						2024					
	Transplanted			Direct seeded rice			Transplanted			Direct seeded rice		
	CF	SA	AWD	CF	SA	AWD	CF	SA	AWD	CF	SA	AWD
First ploughing with disc plough	14 May, 2023	14 May, 2023	14 May, 2023	14 May, 2023	14 May, 2023	14 May, 2023	3 May, 2023	3 May, 2024	3 May, 2024	3 May, 2024	3 May, 2024	3 May, 2024
1st harrowing	17 May, 2023	17 May, 2023	17 May, 2023	17 May, 2023	17 May, 2023	17 May, 2023	10 May, 2023	10 May, 2024	10 May, 2024	10 May, 2024	10 May, 2024	10 May, 2024
2nd harrowing	21 May, 2023	21 May, 2023	21 May, 2023	21 May, 2023	21 May, 2023	21 May, 2023	13 May, 2024	13 May, 2024	13 May, 2024	13 May, 2024	13 May, 2024	13 May, 2024
Planking	21 May, 2023	21 May, 2023	21 May, 2023	21 May, 2023	21 May, 2023	21 May, 2023	13 May, 2024	13 May, 2024	13 May, 2024	13 May, 2024	13 May, 2024	13 May, 2024
Nursery sowing for transplanted rice	22 May, 2023	22 May, 2023	22 May, 2023	–	–	–	15 May, 2024	15 May, 2024	15 May, 2024	–	–	–
Layout and sowing of DSR	–	–	–	22 May, 2023	22 May, 2023	22 May, 2023	–	–	–	15 May, 2024	15 May, 2024	15 May, 2024
Seed rate	60 kg ha^− 1^			23 kg ha^− 1^			60 kg ha^− 1^			23 kg ha^− 1^		
Fertilizer for nursery	2:1:0.5 kg of N: P_2_O_5_:K_2_O per 100 m^2^			–			2:1:0.5 kg of N: P_2_O_5_:K_2_O per 100 m^2^			–		
Fertilizer for main field	120:60:30 kg of N: P_2_O_5_:K_2_O per hectare			120:60:30 kg of N: P_2_O_5_:K_2_O per hectare			120:60:30 kg of N: P_2_O_5_:K_2_O per hectare			120:60:30 kg of N: P_2_O_5_:K_2_O per hectare		
Puddling	20 June, 2023			–			12 June,2024					
Transplanting	20 June, 2023			–			12 June,2024			–		
Hebicide application (pyrazosulfuron ethy + pretilachlor for transplanted rice and triafamon + ethoxysulfuran for DSR)	22 June, 2023			14 June,2023			14 June,2024			12 June,2024		
Harvesting	2 October, 2023			24 September, 2023			28 September, 2024			19 September, 2024		

CF = conventional flooding; SA = saturation; AWD =  alternate wetting and drying; DSR = direct seeded rice.

## Results

### Weather

The mean minimum and maximum temperatures during the crop season were lower in 2023 (12.12 °C and 25.9 °C) as compared to 2024 (13.4 °C and 29.3 °C), respectively (Fig. 1). A difference of 1.28 °C and 3.4 °C was recorded in the mean minimum and maximum temperature, respectively. Rainfall was higher in crop season of 2023 (647.3 mm) as compared 2024 (243.9 mm). Maximum rainfall was recorded in June (92.6 mm) and July (206.6 mm) in year 2023, which coincided with effective tillering and PI stage. In 2024 very less rainfall was received in June and July and maximum rainfall was received in August, which coincided with booting and heading stages of crop.

### Soil analysis

Soil of the experimental site was silty clay loam in texture having 21% sand, and 35% clay. Soil pH, electric conductivity, available N, available P, available K and organic matter were 6.6, 0.45 dsm^− 1^, 273.3 kg ha^− 1^, 16.21 kg ha^− 1^, 268 kg ha^− 1^ and 1.2%, respectively.

### Crop growth

**Fig. 2:**
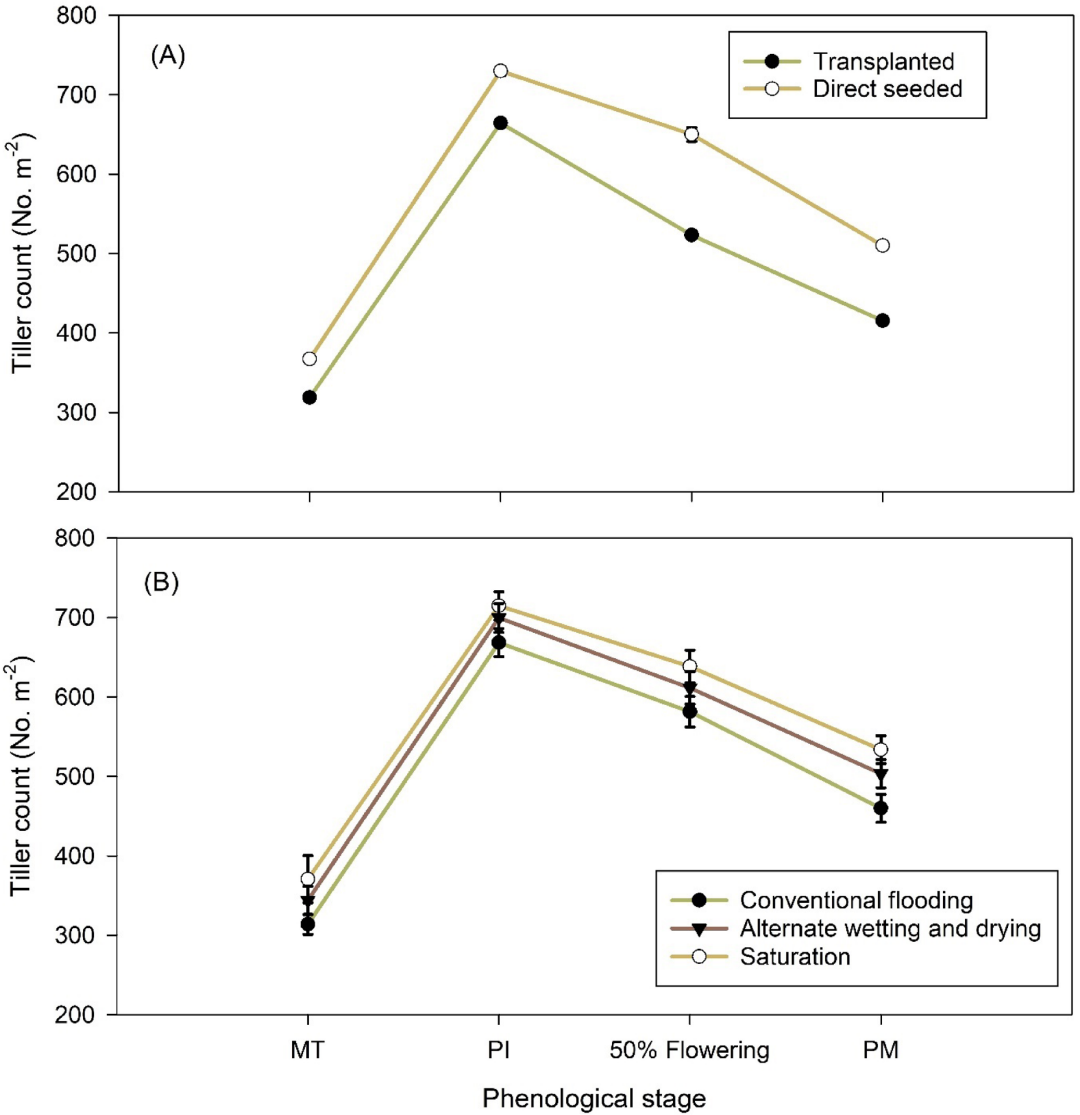
Tiller count in 2023 under two establishment methods (**A**) and three irrigation methods (**B**) in rice. MT =  maximum tillering; PI = panicle initiation; Flow =  flowering; PM = physiological maturity.

Plant height was higher for DSR (132.35 & 132.88 cm) in comparison to transplanted rice (128.46 & 130.52 cm) during both the years of study (Table 2). The difference in plant height however was not significant during both the years. Irrigation methods did not show any significant effect on plant height and the values slightly varied among treatments during both the years. Tiller count increased from tillering to PI stage and recorded a decline towards physiological maturity (Fig. 2). Tiller count was higher in DSR as compared to transplanted rice (Fig. 2A; Table 2) during both the years, respectively. The difference, however, was significant during 2023 with DSR registering 24.3% higher tiller count than transplanted rice at 50% flowering stage. Difference in tiller count was not found significant during 2024. Tiller count did not change significantly with irrigation methods during both years. Interaction was non-significant regarding any of the growth parameters recorded during the study (Table 2).

### Yield attributing traits and crop yield

Yield attributing traits and crop yields were significantly influenced by crop establishment method in 2023 (Tables 2 and 5). Panicle number was higher in DSR (510 & 529 m^−2^) as compared to transplanted rice (415 & 469 m^−2^) during both the years, respectively. Panicle number was 22.9% and 12.7% higher in DSR as compared to transplanted rice during 2023 and 2024, respectively. Difference in panicle number between transplanted and DSR was not significant in 2024. Although numerically higher values for panicle number were obtained under saturation (488 & 534 m^−2^), but the differences were non-significant among different irrigation methods. A reverse situation was observed regarding the number of grains per panicle, which were significantly higher in transplanted rice (101.4) as compared to DSR (96.8) during 2023. The values remained at par during 2024 though numerically higher values were registered in transplanted rice (Table 2). Irrigation methods did not show any significant effect on grains panicle^−1^. The 1000-grain weight was not significantly influenced by either crop establishment or the irritation method during both the years of study. A significantly higher spikelet sterility percentage (12.14%) was noticed in transplanted rice during 2023, and it was non-significant in 2024. Irrigation methods did not influence the sterility percentage significantly, although comparatively higher sterility was observed in CF. During both the years, no significant effect of interaction was observed on yield attributes.

Grain yield, straw yield and carbohydrate equivalent were influenced by crop establishment methods in 2023. Higher grain yield (6.78 & 6.69 t ha^−1^) was registered in DSR as compared to transplanted rice (5.59 & 6.18 t ha^−1^) during 2023 and 2024, respectively. Yield difference was significant only in 2023. Direct seeded rice registered a yield advantage of 21% and 8% over transplanted rice during 2023 and 2024, respectively. Similar response was observed in case of carbohydrate equivalent, where DSR resulted in 0.4–0.9 t ha^−1^ higher carbohydrate equivalent than transplanted rice, in the two years. This indicates that DSR might be more efficient in producing carbohydrates, potentially leading to better yields and higher energy output from the same area of land. Straw yield was 7–8% higher than transplanted rice, in the two years, respectively. Though irrigation methods failed to significantly influence on crop yield, numerically higher values for grain yield, carbohydrate equivalent and straw yield were recorded under saturation method of irrigation. The interaction effect of establishment and irrigation methods was not statistically significant, but the highest grain and straw yield was recorded in DSR with saturation method of irrigation.

### Relationship between yield attributing traits and grain yield

**Fig. 3:**
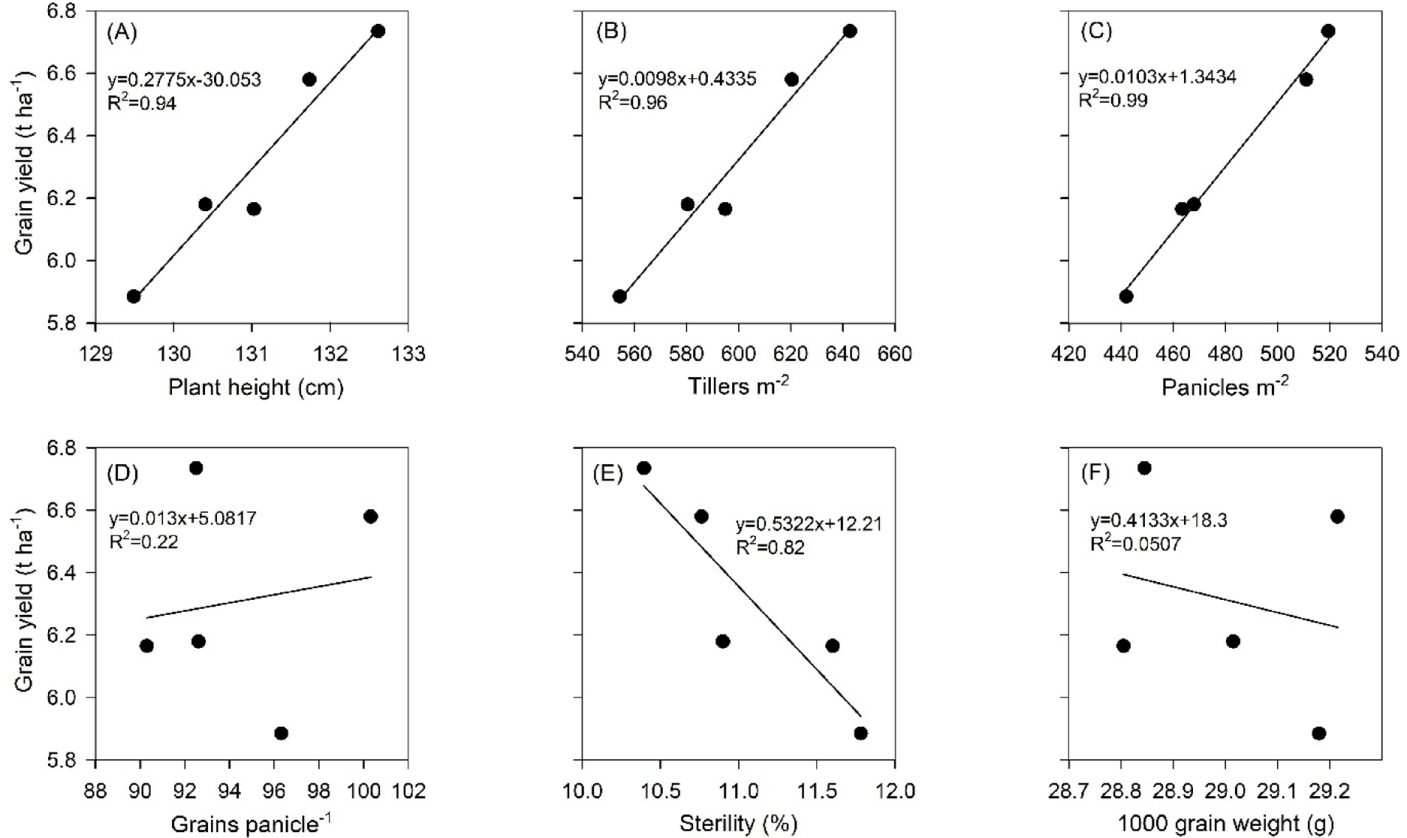
Relationship between plant height (**A**), number of tillers m^− 2^ (**B**), number of panicles m^− 2^ (**C**), number of grains panicle^− 1^ (**D**), sterility (**E**) and 1000 grain weight (**F**) with grain yield. Black dots represent the average of two-year data and solid line represents the linear regression fit for the specified parameters.

Growth parameters such as plant height (Fig. 3A) and tiller count (Fig. 3B) depicted a linear relation (R^2^ = 0.94 & 0.96) with grain yield, respectively. Among the yield attributing traits, panicle number (Fig. 3C) showed a strong linear relation (R^2^ = 0.99) to grain yield. A weak relation was found between grains panicle^− 1^ (R^2^ = 0.22) (Fig. 3D) and 1000 grain weight (R^2^ = 0.05) (Fig. 3F) with grain yield. A strong negative relation between spikelet sterility and grain yield (R^2^ = 0.82) was observed (Fig. 3E).

**Table 2:** Crop growth and yield attributes as influenced by different establishment methods and irrigation regimes.

Treatments	Plant height (cm) at Physiological Maturity		Tiller count m^− 2^ (At flowering)		Panicles m^− 2^		Grains panicle^− 1^		Spikelet sterility (%)		1000 grain weight (g)	
	2023	2024	2023	2024	2023	2024	2023	2024	2023	2024	2023	2024
Establishment methods												
Transplanted rice	128	130	523	586	415	469	101	91	12.0	11.4	29.06	29.30
DSR	132	133	650	635	510	529	97	88	9.9	10.8	28.53	29.16
SEm±	0.66	1.26	27.4	13.35	24.6	17.84	1.12	0.49	0.27	0.27	0.412	0.36
CD (*P* = 0.05)	NS	NS	102.3	NS	86.8	NS	3.83	NS	0.89	NS	NS	NS
Irrigation methods												
CF	131	131	608	581	467	459	98	82	12.0	11.2	28.43	29.18
SA	131	133	603	638	488	534	103	97	10.6	10.9	29.03	29.40
AWD	129	132	549	611	432	504	96	89	10.5	11.2	28.91	29.12
SEm±	0.80	0.95	26.00	21.33	20.15	28.61	5.64	2.96	0.36	0.27	0.455	0.351
CD (*P* = 0.05)	NS	NS	NS	NS	NS	NS	NS	NS	NS	NS	NS	NS
Establishment × Irrigation methods												
Transplanted × CF	129	128	524	544	408	428	101	85	13.5	11.5	29.17	29.5
Transplanted ×SA	128	132	570	618	462	508	109	96	11.7	11.2	28.93	29.4
Transplanted × AWD	128	131	475	595	374	471	94	92	11.3	11.6	29.07	29
SEm±	1.15	2.18	61.48	23.12	60.56	30.90	9.23	0.85	0.62	0.46	0.714	0.624
CD (*P* = 0.05)	NS	NS	NS	NS	NS	NS	NS	NS		NS	NS	NS
DSR × CF	134	133	693	619	527	491	95	80	10.6	10.9	27.70	28.87
DSR × SA	133	134	635	658	514	560	98	98	9.5	10.7	29.13	29.40
DSR × AWD	130	132	624	627	490	536	98	87	9.8	10.9	28.76	29.23
SEm±	1.14	1.67	46.49	28.01	42.00	37.54	8.41	3.45	0.47	0.41	0.668	0.543
CD (*P* = 0.05)	NS	NS	NS	NS	NS	NS	NS	NS	NS	NS	NS	NS

CF = conventional flooding; SA = saturation; AWD = alternate wetting and drying; DSR = direct seeded rice.

### Crop phenology

**Fig. 4:**
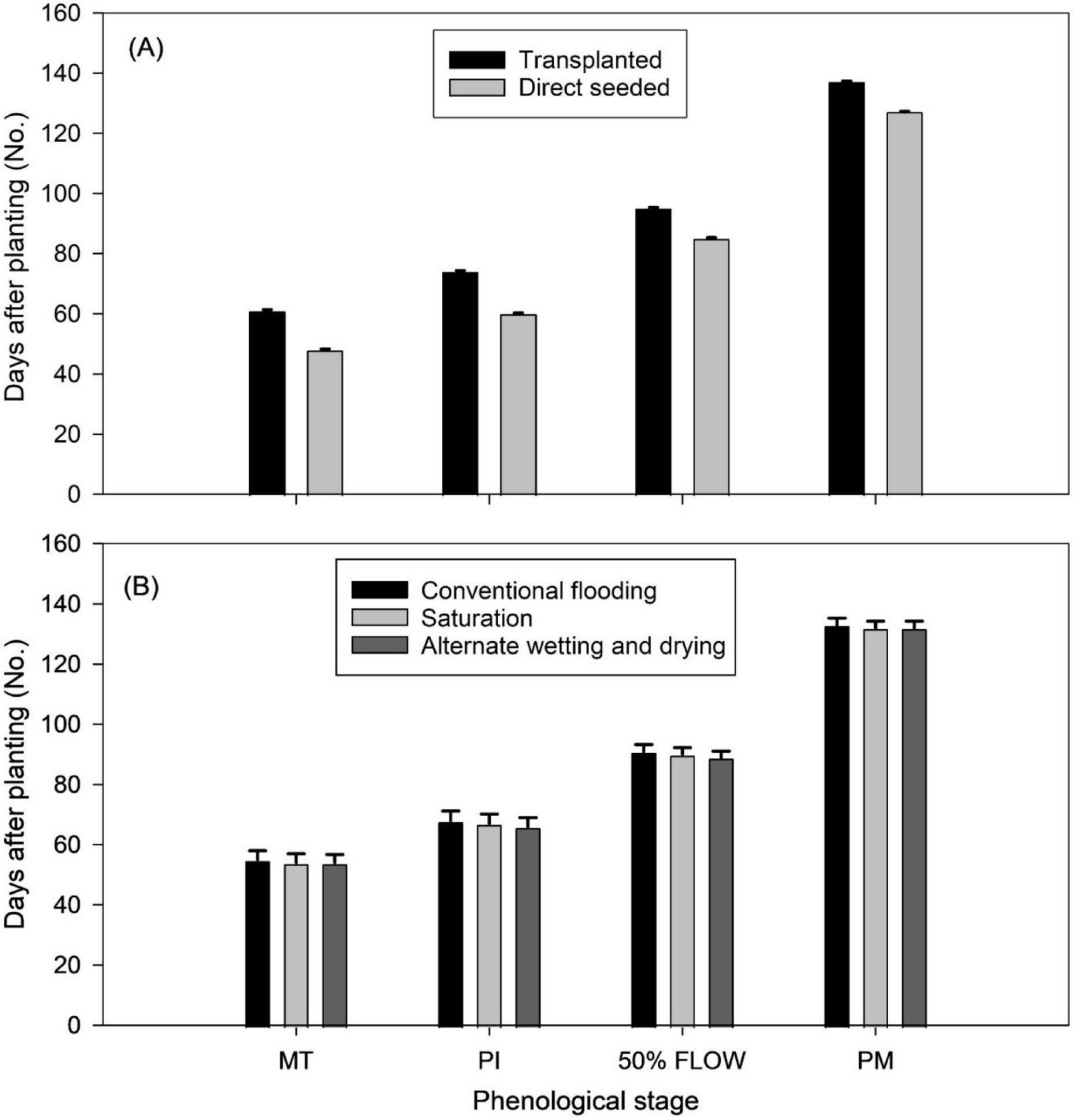
Phenological stages of rice under two establishment (**A**) and three irrigation methods (**B**). (**A**) is the mean across three irrigation methods and (**B**) is the mean across two establishment methods. MT = maximum tillering; PI = panicle initiation; Flow = flowering; PM = physiological maturity.

Crop phenology stages such as maximum tillering, PI, 50% flowering and physiological maturity were substantially influenced by the establishment methods (Fig. 4A). The respective stages reached 12.6, 13.6, 9.6 and 10 days earlier in DSR as compared to transplanted rice. Crop phenology was not influenced by irrigation methods (Fig. 4B). Crop reached maturity in 127 and 137 days after sowing in DSR and transplanted rice, respectively. This indicates a 10-day earlier maturity in DSR.

### Agro-climatic indices

Growing degree days and HTUs accumulated at different phenological stages varied among establishment methods and Irrigation methods (Table 3). Irrespective of treatments, the accumulated GDD and HTU increased from early growth stage towards crop maturity. Direct seeded rice accumulated significantly less GDDs and HTUs at each phenological stages as compared to transplanted rice. Total GDDs and HTUs accumulated by DSR were 1224–1274 (°C day) and 10238–10319 (°C day h) in 2023 and 2024, respectively. In transplanted rice 1466–1467 (°C day) and 11686–12154 (°C day h) GDDs and HTU were recorded in 2023 and 2024, respectively. Among the irrigation methods, GDDs and HTUs were significantly higher in CF. Heat use efficiency also varied significantly under different establishment methods with higher values of 5.32–5.47 (kg ha^−1^ °C day) registered in DSR and 3.81–4.21 (kg ha^−1^ °C day) recorded in transplanted rice. HUE was at par among irrigation methods with slightly lower in CF than saturation and AWD.

**Table 3:** Agro-climatic indices as influenced by establishment and irrigation method.

Treatments	GDD (°C day) Maximum Tillering		HTU (°C day h) Maximum Tillering		GDD (°C day) Panicle initiation		HTU (°C day h) Panicle initiation		GDD (°C day) 50% flowering		HTU (°C day h) 50% flowering		GDD (°C day) Physiological maturity		HTU (°C day h) Physiological maturity		HUE (kg ha^− 1^ °C day)	
	2023	2024	2023	2024	2023	2024	2023	2024	2023	2024	2023	2024	2023	2024	2023	2024	2023	2024
Establishment methods																		
Transplanted	647	763	4868	6958	814	855	6071	7797	1120	1168	8720	10,019	1466	1467	11,686	12,154	3.81	4.21
DSR	486	575	3771	5514	639	657	4868	5931	979	1019	7640	8900	1274	1224	10,238	10,319	5.32	5.47
SEm±	0.236	0.172	0.75	4.25	0.116	0.334	1.853	0.97	0.927	0.548	23.27	4.508	0.973	0.208	31.769	0.614	0.051	0.116
CD (*P* = 0.05)	1.543	1.127	4.915	27.843	0.762	2.191	12.137	6.352	6.074	3.591	152.47	29.531	6.374	1.362	208.12	4.02	0.337	0.758
Irrigation methods																		
CF	577	675	4346	6285	738	761	5530	6910	1061	1104	8275	9519	1381	1356	11,088	11,296	4.48	4.54
SA	565	667	4306	6212	724	754	5478	6841	1048	1089	8134	9430	1369	1340	10,899	11,207	4.75	4.97
AWD	558	667	4306	6212	717	754	5400	6841	1041	1089	8134	9430	1361	1340	10,899	11,207	4.31	4.84
SEm±	0.408	0.097	1.235	0.148	0.197	0.115	0.952	0.506	0.647	0.723	29.02	1.299	0.326	0.152	38.905	0.684	0.126	0.129
CD (*P* = 0.05)	0.564	0.322	4.091	0.492	0.653	0.379	3.152	1.674	1.191	2.396	96.12	4.303	1.08	0.504	128.845	2.265	NS	NS

GDD = growing degree days; HTU = heat use efficiency; CF = conventional flooding; SA = saturation; AWD = alternate wetting and drying.

### Water productivity

**Table 4:** Total water input and water saving under two establishment and three irrigation methods.

Treatment	Water input (mm)						Water saving over transplanted/ CF (%)	
	2023			2024			2023	2024
	Irrigation	Rainfall	Total	Irrigation	Rainfall	Total		
Establishment methods								
Transplanted rice	1207	647.3	1854	1425	243.9	1669	–	–
DSR	1159	647.3	1806	1375	243.9	1619	2.58	3.0
SEm±	1.848	–	2.192	3.624	–	3.72	–	–
CD (*P* = 0.05)	12.104	–	14.36	23.74	–	24.38	–	–
Irrigation methods								
CF	1528	647.3	2175	1728	243.9	1972	–	–
SA	1028	647.3	1675	1243	243.9	1487	23.0	24.5
AWD	993	647.3	1640	1228	243.9	1472	24.6	25.3
SEm±	4.575	–	4.563	10.53	–	10.5	–	–
CD (*P* = 0.05)	15.15	–	15.11	34.89	–	34.9	–	–
Establishment × Irrigation methods								
Transplanted × CF	1578	647.3	2225	1729	243.9	2011	–	
Transplanted × SA	1041	647.3	1688	1244	243.9	1499	24.13	25.4
Transplanted × AWD	1001	647.3	1648	1228	243.9	1498	25.9	25.5
SEm±	3.2	–	3.79	6.28	–	6.44	–	–
CD (*P* = 0.05)	23.714	–	24.46	NS	–	NS	–	–
DSR × CF	1478	647.3	2126	1729	243.9	1934	–	–
DSR x SA	1015	647.3	1662	1244	243.9	1476	21.8	23.6
DSR x AWD	985	647.3	1632	1228	243.9	1446	23.2	23.2
SEm±	5.597	–	5.70	12.69	–	12.71	–	–
CD (*P* = 0.05)	20.512	–	21.63	NS	–	NS	–	–

CF = conventional flooding; SA = saturation; AWD = alternate wetting and drying.

Water inputs including the rainfall and irrigation water under different treatments is given in Table 4. We found a substantial variation in water inputs between two years of study. Irrigation water input was 15.49% less in 2023 than 2024. Establishment and Irrigation methods showed significant difference in water input with Transplanted rice consuming more water than DSR. Among the irrigation methods, CF consuming an additional 500 mm and 535 mm than SA and AWD in 2023, respectively. In 2024, CF consumed 484 mm and 500 mm greater than SA and AWD, respectively. Actual water saving, including rainfall and irrigation water, was 23–24.5% and 24.6 to 25.3% in saturation and AWD over CF (Table 4). Interaction was also found significant with transplanting x CF consuming significantly higher volume (2011–2225 mm) of water.

Among establishment methods, DSR recorded significantly higher WP (0.38 & 0.42 kg m^−3^) as compared to transplanted rice (0.31 & 0.38 kg m^−3^) during 2023 and 2024, respectively (Table 5). Irrigation methods produced a significant effect on WP. On an average CF had 28% and 25% lesser WP than saturation and AWD, respectively.

### Economics

Maximum profit of ₹ (Indian rupee) 2.2 per ₹ invested was obtained under DSR as compared to transplanted rice, in which B: C reduced due to higher transplanting costs (Table 5). Among the irrigation methods, B:C did not vary substantially between the saturation and the AWD method but was higher than the CF. Among interactions, DSR × saturation or DSR × AWD was superior in B: C to DSR x CF and with all irrigation methods under transplanted method of establishment. This indicates economic superiority of DSR over transplanted rice.

## Discussion

### Crop growth

Our study shows that plant growth traits were better in DSR as compared to transplanted rice, particularly in year 2023. This variation may be attributed to difference in weather conditions between the two years. Additionally, sowing was relatively delayed in 2023, which in turn postponed transplanting in transplanted rice. This delay likely slowed crop development and exposed the crop to less favourable environmental conditions, ultimately reducing yield. In contrast, the absence of transplanting shock in DSR may have allowed the crop to reach key phenophases earlier, aligning with favourable environmental conditions.

Earlier^29^ also reported significance of time of sowing and transplanting of rice for achieving better growth and yield in rice. Earlier studies also reveal better crop growth in DSR than transplanted rice owing to absence of transplanting shock^13^ and better seedling growth in DSR^30^enabling it to utilize resources efficiently^31^. Our study also shows that irrigation methods did not produce significant effect on crop growth either as main treatment or in the interaction with DSR and transplanted rice. Our results match with those of^13^who reported that irrigation methods did not significantly impact important growth parameters.

### Yield attributing traits and crop yield

Yield attributing traits such as panicles m^− 2^, grains panicle^− 1^, 1000 grain weight and spikelet sterility varied between transplanted rice and DSR. Panicles m^− 2^ were significantly higher in DSR in comparison to transplanted rice in 2023, which might be due to better crop vigour in the DSR and exposure of growth stages of transplanted rice to less favourable environment resulting from delayed nursery sowing and main field transplanting in 2023. Although grains panicle^− 1^ were significantly lower in DSR than transplanted rice, yield was higher in DSR. This might be due to significantly higher panicles m^− 2^, which had a stronger linear relation with grain yield, compared to grains panicle^− 1^ (Fig. 3C). Significantly higher spikelet sterility was recorded in transplanted rice in comparison to DSR during 2023, but in 2024 the effect was non–significant. This may be attributed to the late transplanting of the crop in 2023, which likely prolonged phenophases due to transplanting shock and exposed the flowering period to unfavourable temperature. Direct seeded rice on the other side attained growth stages faster as it skips additional time required for raising of seedlings in nursery and avoided uprooting/ transplanting shock^32^. A meta-analysis of seven-year data between 2010 and 2017 by^1^ indicates that panicle number has a positive and significant response to direct seeding of rice, whereas the spikelet number per panicle had a negative response.

We obtained higher yield in DSR as compared to transplanted rice. This might be due to higher number of tillers, higher panicles m^− 2^ and lower spikelet sterility in DSR than transplanted rice (Table 2). A linear response analysis indicated that grain yields was strongly and positively related to plant height (R^2^ = 0.94, Fig. 3A), number of tillers m^− 2^ (R^2^ = 0.96, Fig. 3B) and panicles m^− 2^ (R^2^ = 0.99, Fig. 3C), and negatively related to spikelet sterility (R^2^ = 0.83, Fig. 3E). All these yield attributes were higher in DSR than the transplanted rice, together with lower spikelet sterility. Both grains panicle^− 1^ and 1000-grain weight were higher in transplanted rice than DSR (Table 2), but the response analysis indicated that these were not strongly related to yield (R^2^ = 0.22 − 0.05, Fig. 3D and 3F)^13^. reported that yield advantage of DSR over transplanted rice was due to higher number of panicles m^− 2^. Our results agree to those of^12,33^who showed that transplanted rice is not superior to well managed DSR.

**Table 5:** Yield, carbohydrate equivalent, water productivity and benefit cost ratio (B: C) as influenced by different crop establishment and irrigation methods.

Treatments	Grain yield (t ha^− 1^)		Straw yield (t ha^− 1^)		Carbohydrate equivalent (t ha^− 1^)		Water productivity (kg m^− 3^)		B: C	
	2023	2024	2023	2024	2023	2024	2023	2024	2023	2024
Establishment methods										
Transplanted rice	5.59	6.18	7.17	7.07	4.36	4.82	0.31	0.38	1.47	1.50
DSR	6.78	6.69	7.69	7.61	5.29	5.22	0.38	0.42	2.20	2.21
SEm±	0.07	0.15	0.154	0.15	0.06	0.11	0.009	0.011	–	–
CD (*P* = 0.05)	0.49	NS	0.508	NS	0.38	NS	NS	NS	–	–
Irrigation methods										
CF	6.18	6.15	7.425	7.02	4.82	4.8	0.28	0.31	1.84	1.75
SA	6.50	6.66	7.908	7.55	5.07	5.20	0.39	0.45	1.93	1.90
AWD	5.87	6.49	6.953	7.44	4.57	5.07	0.36	0.44	1.74	1.91
SEm±	0.15	0.18	0.29	0.23	0.17	0.14	0.008	0.011	–	–
CD (*P* = 0.05)	NS	NS	NS	NS	NS	NS	0.03	0.038	–	–
Establishment × Irrigation method										
Transplanted × CF	5.77	5.81	7.56	6.68	4.5	4.54	0.26	0.29	1.55	1.38
Transplanted × SA	5.70	6.47	7.29	7.30	4.45	5.04	0.34	0.43	1.47	1.57
Transplanted × AWD	5.30	6.27	6.66	7.22	4.14	4.89	0.321	0.42	1.39	1.56
SEm±	0.13	0.25	0.28	0.27	0.10	0.20	0.006	0.019	–	–
CD (*P* = 0.05)	NS	NS	NS	NS	NS	NS	NS	NS	–	–
DSR × CF	6.60	6.49	7.29	7.37	5.15	5.06	0.31	0.34	2.13	2.11
DSR × SA	7.30	6.86	8.52	7.79	5.70	5.36	0.44	0.46	2.39	2.23
DSR × AWD	6.43	6.72	7.25	7.65	5.02	5.24	0.39	0.46	2.10	2.27
SEm±	0.19	0.25	0.28	0.30	0.15	0.20	0.011	0.017	–	–
CD (*P* = 0.05)	NS	NS	NS	NS	NS	NS	NS	NS	–	–

CF = conventional flooding; SA = saturation; AWD = alternate wetting and drying; DSR = direct seeded rice; SEm = standard error of mean; CD = critical difference; B:C = benefit-cost ratio.

Absence of significant effect of irrigation methods on yield attributes and grain yield suggests that saturation and AWD method were able to sustain higher yields and there is no need for CF, which diminishes WP. Our results match with the findings of^34^who found no difference in rice yield attributes and grain yield under CF and AWD.

### Crop phenology

Crop phenophases were achieved earlier in DSR compared to transplanted rice. On an average there was a difference of 10 days between the maturity of crop under DSR and transplanted rice. This might be attributed to transplanting shock in the later, which inhibited early seedling growth^35^. Previous studies also confirm early maturity in DSR compared to transplanted rice^13,36,37^.

### Agro-climatic indices

We found that DSR accumulated lesser GDDs and HTUs to reach different phenophases. This may be attributed to lack of transplanting shock, consequently requiring less days to reach maturity^38^. This particularly has a great significance under temperate climatic conditions where seasons are comparatively short. Our results also indicate that HUE was higher in DSR in comparison to transplanted rice. This might be due to higher yields attained in comparatively shorter duration in DSR as compared to transplanted rice. Under similar environment^25^, also reported higher HUE in treatments attaining higher yields in shorter duration and vice-versa.

### Water input and water productivity

Water input through irrigation was higher in 2024 in comparison to 2023. This might be attributed to less rainfall and high temperature in 2024, which might have increased evapotranspiration demand of crop. Total water input including rainfall and irrigation however was higher in 2023. This was due to heavy rainfall in June and July 2023, a major portion of which was not utilized by crop. Owing to nursery raising and puddling operation, water input was higher in transplanted rice than DSR. For the same reason and because of higher yields, water productivity in DSR was greater than transplanted rice. Additionally, reduced crop duration in DSR decreased water requirement and improved WP^39^. Previously^40,41^, also reported water saving and higher WP in DSR. As expected, irrigation methods produced a significant effect on WP with CF recording decrease in WP than saturation and AWD. According to^42^CF is less efficient in water saving owing to increased seepage and percolation, puddling and evaporative losses.

### Economics

Returns per rupee invested were higher in DSR as compared to transplanted rice. This was due to higher productivity^25^ and comparatively lower inputs for cultivation of DSR^43^. Transplanted rice needs additional resources for nursery raising, uprooting, seedling transportation to main field, puddling and transplanting of seedlings in main field. This increases cost of cultivation and decreases profitability of transplanted rice as compared to DSR.

## Conclusion

Direct-seeded rice (DSR) cultivation presents a viable alternative amid rising input costs and dwindling water resources. When managed effectively, DSR can lead to improved crop growth, higher yields, and better economic returns compared to traditional transplanted rice. In our study, DSR demonstrated a yield advantage of 8–21% and enhanced water productivity by 11–25% over transplanted rice. Additionally, both saturated and alternate wetting and drying irrigation methods produced yields comparable to conventional flooding, while significantly reducing water usage. These findings highlight DSR, combined with saturated or AWD irrigation, as an efficient and sustainable option for rice cultivation.

## Data Availability

The datasets used and/or analyzed during the current study are available from the corresponding author on reasonable request.
